# Automated detection and classification of osteolytic lesions in panoramic radiographs using CNNs and vision transformers

**DOI:** 10.1186/s12903-025-06209-6

**Published:** 2025-06-21

**Authors:** Niels van Nistelrooij, Iman Ghanad, Amir K. Bigdeli, Daniel G. E. Thiem, Constantin von See, Carsten Rendenbach, Ira Maistreli, Tong Xi, Stefaan Bergé, Max Heiland, Shankeeth Vinayahalingam, Robert Gaudin

**Affiliations:** 1 Department of Oral and Maxillofacial Surgery, Radboud University Medical Center, P.O. Box 9101, Nijmegen, 6500 HB the Netherlands; 2 Department of Oral and Maxillofacial Surgery, Charité - Universitätsmedizin Berlin, corporate member of Freie Universität Berlin and Humboldt Universität zu Berlin, Augustenburger Platz 1, Berlin, 13353 Germany; 3 Department of Hand, Plastic and Reconstructive Surgery, BG Trauma Center Ludwigshafen, University of Heidelberg, Burn CenterLudwig-Guttmann-Strasse 13, Ludwigshafen, 67071 Germany; 4 Department of Oral and Maxillofacial Surgery, University Medical Centre, Johannes Gutenberg University Mainz, Augustusplatz 2, Mainz, 55131 Germany; 5 Department of Dentistry, Faculty of Medicine and Dentistry, Danube Private University, Steiner Landstrasse 124, Krems an Der Donau, 3500 Austria

**Keywords:** Deep Learning, Osteolytic Lesions, Panoramic Radiograph, Vision Transformer

## Abstract

**Background:**

Diseases underlying osteolytic lesions in jaws are characterized by the absorption of bone tissue and are often asymptomatic, delaying their diagnosis. Well-defined lesions (benign cyst-like lesions) and ill-defined lesions (osteomyelitis or malignancy) can be detected early in a panoramic radiograph (PR) by an experienced examiner, but most dentists lack appropriate training. To support dentists, this study aimed to develop and evaluate deep learning models for the detection of osteolytic lesions in PRs.

**Methods:**

A dataset of 676 PRs (165 well-defined, 181 ill-defined, 330 control) was collected from the Department of Oral and Maxillofacial Surgery at Charité Berlin, Germany. The osteolytic lesions were pixel-wise segmented and labeled as well-defined or ill-defined. Four model architectures for instance segmentation (Mask R-CNN with a Swin-Tiny or ResNet-50 backbone, Mask DINO, and YOLOv5) were employed with five-fold cross-validation. Their effectiveness was evaluated with sensitivity, specificity, F1-score, and AUC and failure cases were shown.

**Results:**

Mask R-CNN with a Swin-Tiny backbone was most effective (well-defined F1 = 0.784, AUC = 0.881; ill-defined F1 = 0.904, AUC = 0.971) and the model architectures including vision transformer components were more effective than those without. Model mistakes were observed around the maxillary sinus, at tooth extraction sites, and for radiolucent bands.

**Conclusions:**

Promising deep learning models were developed for the detection of osteolytic lesions in PRs, particularly those with vision transformer components (Mask R-CNN with Swin-Tiny and Mask DINO). These results underline the potential of vision transformers for enhancing the automated detection of osteolytic lesions, offering a significant improvement over traditional deep learning models.

## Background

Osteolytic lesions occurring in jaws are characterized by the gradual absorption of bone tissue, typically evident as radiolucent areas in radiographic imaging [1, 2]. Patients with early-stage osteolytic lesions are often asymptomatic, allowing the underlying disease to progress considerably before it is diagnosed due to symptoms such as pain, swelling, or fracture [3, 4]. Diseases associated with osteolytic lesions include microbial infections, avascular osteonecrosis and osteomyelitis. Furthermore, malignant diseases such as metastasized cancer or multiple myeloma, accounting for 15% of hematologic malignancies [5–9] , are frequently associated with osteolytic processes.

Detecting osteolytic lesions at an early stage has always been a challenge due to lack of clinical symptoms [10]. Moreover, each underlying disease associated with an osteolytic lesion exhibits distinctive radiographic patterns, which can be categorized into two groups [6]: well-defined osteolytic lesions with a sharp border (indicative of benign cyst-like lesions) and ill-defined osteolytic lesions with an unclear border and occasionally spiculation (indicative of osteomyelitis or malignant lesions) [11]. Therefore, obtaining a complete overview through a computed tomography (CT) scan, cone-beam computed tomography (CBCT) scan or panoramic radiograph (PR) is important for examining and ruling out osteolytic lesions [1, 12]. A PR is often preferred over a (CB)CT scan due to its shorter acquisition time and lower radiation dose [13, 14].

Patients clearly benefit from an early detection of osteolytic lesions because it allows for timely intervention to limit further progression of the disease and to avoid more severe complications. However, pathological structures can be easily overlooked on a PR during routine clinical examination. Additionally, PRs often display artifacts as well as radiopaque and radiolucent spots that represent various structures in the examined areas, along with shadows from soft tissues and anatomical air spaces. To accurately interpret the PR, a dentist must be knowledgeable in these areas. However, even if a dentist is experienced and has a lot of radiological knowledge, automated detection of the respective pathologies could be a significant aid to improve detection accuracy.

Recent studies suggest that artificial intelligence (AI) assisted methods for diagnosing dental pathologies on PRs using convolutional neural networks (CNNs) are at least as effective as experienced dentists [15]. Prior research has illustrated the effective analysis of PRs using AI for the segmentation of teeth [16], detection of fractures [17], periodontal bone loss [18], and caries lesions [19]. The most recent AI methods in the dental field are based on vision transformers, resulting in improved effectiveness for detection of pathologies on PRs compared to CNNs [20]. Vision transformers treat images as sequences of tokens and learn dependencies among them. This enables the modelling of long-range patterns in images, making them highly effective for super-resolution tasks. The application of AI methods for medical tasks involving osteolytic lesions currently follows two distinct directions. The first direction analyzes whole-body CT scans for detection and classification of malignant osteolytic lesions, with a focus on spinal osteolytic lesions [21–23]. The second direction applies CNNs for detection and classification of well-defined osteolytic lesions on PRs [24–31].

Previous studies have primarily focused on detecting odontogenic cysts and lesions using cropped images or limited fields of view from cone-beam computed tomography (CBCT), our study uses a novrl approach with visual transformers to detect well-defined and ill-defined osteolytic lesions directly on PRs [30, 32, 33].

Current research has not yet investigated the application of AI methods for the detection of ill-defined osteolytic lesions on PRs, which could indicate osteomyelitis or malignant jaw lesions. Moreover, AI methods based on CNNs or vision transformers can assist a clinician in improving their sensitivity and guide dentists to areas requiring closer evaluation. Therefore, the current study aimed to detect osteolytic lesions in PRs and classify them as well-defined or ill-defined using CNNs and vision transformers.

## Methods

The checklist for artificial intelligence (AI) research in dentistry has been reviewed for reporting [34].

### Data

Six thousand four hundred four panoramic radiographs (346 PRs with osteolytic lesion, 6058 PRs without) were randomly collected and retrospectively included in the current study between 2013 and 2023 from the Department of Oral and Maxillofacial Surgery of Charité Berlin, Germany. The included patients had a median age of 42 years, interquartile range (IQR) of 34 years, and an age range of 18–99 years. 3728 patients were male (median age 38 years, IQR 32, age range 18–92 years) and 2438 were female (median age 48 years, IQR 35, age range 18–99 years). A small number of patients had multiple PRs taken for this study. The PRs were obtained with devices from two different manufacturers (Orthophos XG, Sirona and Elite CR, Kodak) and were exported in JPEG format. PRs were excluded if they were blurred, incomplete, or exhibited severe artifacts that impaired diagnostic interpretation. Images taken post-surgical interventions—such as apical resections, extractions, or cyst/tumor surgeries—were not systematically excluded unless the surgical changes compromised the visibility of the jaw structures or interfered with lesion annotation.

### Data annotation

**Fig. 1:**
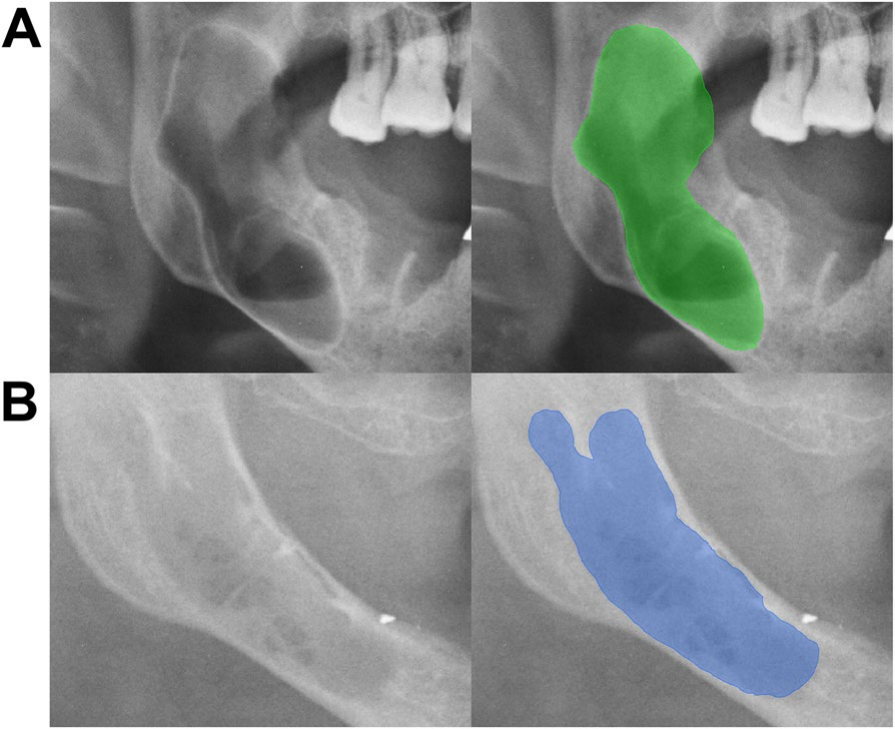
Examples of well-defined and ill defined lesions on PR. Image **A** illustrates a well-defined lesion with sharp and distinct borders. In contrast, image **B** presents an ill-defined lesion characterized by poorly demarcated margins, blending into the surrounding bone and appearing blurry or irregular

The included PRs were annotated for well-defined and ill-defined osteolytic lesions by an experienced board-certified oral surgeon (OS) and a resident in oral and maxillofacial surgery (MS), following training by an oral and maxillofacial radiologist (DT). Ill-defined lesions were characterized by blurred or irregular borders lacking clear demarcation from surrounding bone, while well-defined lesions exhibited sharp, distinct margins clearly separating them from adjacent structures (Fig. 1). Based on electronic medical records (EMR), including clinical and additional radiological examinations, the surgeon located a lesion by drawing a bounding box around the lesion and labeling it as either well-defined or ill-defined. Furthermore, the mandibular sub-regions (median, paramedian, corpus, angle, ramus, coronoid, condyle) and the maxilla were noted if they were affected by an osteolytic lesion to characterize the common locations of osteolytic lesions [35]. Following the development of a preliminary model, the resident reviewed the bounding boxes and annotated each osteolytic lesion as a pixel-level segmentation supported by the model’s predictions to improve sensitivity using the Darwin platform (V7 Labs, London, UK). No cases included both a well-defined and an ill-defined osteolytic lesion. Thus, a PR could be categorized as either control, well-defined or ill-defined.

A random sample of the control PRs was taken to create the final dataset with 330 control, 165 well-defined, and 181 ill-defined PRs. These PRs were divided into five folds with stratification, and five-fold cross-validation splits were determined with 4/1 folds for training and validation, respectively. The training PRs were used to optimize the parameters of the deep learning models and the validation PRs were used for evaluating a model’s effectiveness after training.

### Model architectures

**Fig. 2:**
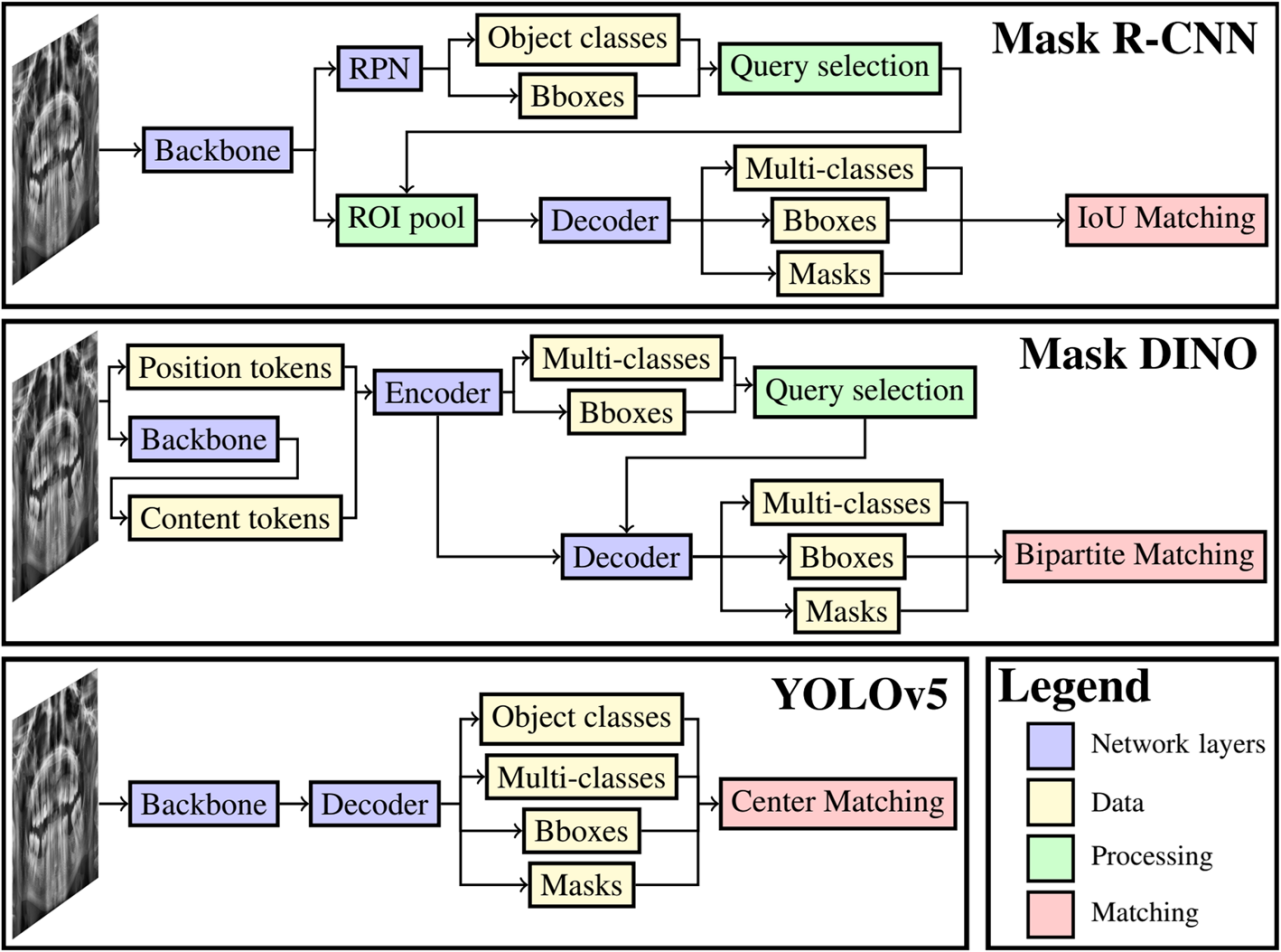
Overview of deep learning models. Mask R-CNN and Mask DINO are multi-stage models as they re-use the features from the backbone and encoder, respectively, whereas YOLOv5 is a one-stage model. Object classes predict whether there exists any object within a bounding box (binary classification) and the multi-classes predict the class of an object in the bounding box. Each model utilizes a different method for matching predicted objects to reference objects to update the model parameters. RPN = region proposal network, ROI = region of interest, IoU = intersection over union

In the current study, four deep learning models for instance segmentation were compared with each other (Fig. 2). Instance segmentation predicts osteolytic lesions as pixel-level segmentations and labels them as ill-defined or well-defined. Two Mask region-based convolutional neural network (R-CNN) combinations (with ResNet-50 and Swin-Tiny) were chosen to represent the first effective method for instance segmentation and a variant with Vision Transformer components. The third model was Mask DINO, chosen for its design that requires Vision Transformer components. The last chosen model was YOLOv5 to represent an effective method for real-time instance segmentation.

*Mask R-CNN* is characterized by a two-stage approach. In the first region-proposal stage, it predicts bounding boxes around all objects from a hand-crafted set of boxes around each pixel. Subsequently, the second stage aggregates the image features within these proposed boxes for object classification, refinement of the bounding boxes, and prediction of foreground segmentations [36]. During training, the model parameters are updated by comparing the reference objects to a balanced sample of boxes predicted to contain an object and boxes predicted not to contain an object. Furthermore, post-processing involves the application of non-maximum suppression (NMS) to select non-overlapping objects. To investigate the distinctions between using a CNN and a vision transformer as the backbone architecture, both ResNet-50 [37] and Swin-Tiny [38] were employed.

*Mask DINO* is an end-to-end instance segmentation approach using vision transformer layers to initialize anchor boxes, as opposed to relying on a hand-crafted set of anchor boxes. The initial anchor boxes are refined a number of times by additional vision transformer layers, after which the final object classes and segmentations are predicted [39]. The model parameters are updated by first determining a bipartite (one-to-one) matching between detected and reference objects. All detected objects were supervised for classification, including whether each object matched a reference object. Conversely, supervision for bounding box refinement and segmentation was done by comparing only the matched detected objects to their corresponding reference objects.

*YOLOv5* is a one-stage approach for instance segmentation in real-time. The model divides an input image into a square grid of cells and predicts bounding boxes, object classes, and segmentations for each cell that contains the center of an object [40]. The model is supervised similarly to Mask R-CNN by comparing the reference objects to a balanced sample of cells predicted to contain the center of an object and cells predicted not to contain an object. Finally, post-processing applies NMS to select non-overlapping objects. The YOLOv5 model uses a CNN backbone architecture specifically designed for object detection and the medium variant of the backbone architecture was used for the current study.

### Model training

A model was first pre-trained on the common objects in context (COCO) dataset, which contains photographs with people, animals, and common objects [41]. Each model was then fine-tuned five times given the training PRs of each cross-validation split (subsection Data annotation). Mask R-CNN and Mask DINO were implemented using the MMDetection framework (v3.0.0), whereas YOLOv5 was implemented using the MMYOLO framework (v0.6.0) [42–44]. The AdamW optimizer was used with a weight decay of 0.05 and an initial learning rate of 1 · 10 − 4 [45]. Mask R-CNN and Mask DINO were run for 36 epochs with a mini-batch size of 2 and the learning rate was divided by 10 after epochs 27 and 33, whereas YOLOv5 was run for 200 epochs with a mini-batch size of 16 and the learning rate was divided by 10 after epochs 150 and 183. PRs were augmented during training with random flipping, resizing, and cropping.

### Model inference

Two scores per PR for a well-defined and ill-defined lesion were determined as the maximum classification probability from all detected well-defined and ill-defined lesions, respectively. Finally, a PR was classified as control if neither lesion score was at least 0.5. Otherwise, the PR’s class followed the maximum lesion score (well-defined or ill-defined). Training and evaluation were performed on a computer with three RTXA600048GB GPU and 128GB memory.

### Statistical analysis

Each fine-tuned model was used to predict lesions in PRs from the model’s respective validation fold. The predicted lesions from each model were accumulated to form predictions for all PRs, which were compared to the reference annotations using scikit-learn (v1.3.0). Classification metrics are reported as sensitivity = TPTP + FN, specificity = TNTN + FP, and F1-score = 2*TP2*TP + FP + FN, where TP, TN, FP, and FN denote true positives, true negatives, false positives, and false negatives, respectively. Furthermore, the receiver-operating-characteristics (ROC) curve and its area under the curve (AUC) are presented for each lesion (well-defined or ill-defined) and model architecture. Moreover, confusion matrices and misclassified predictions are shown to provide a better understanding of the mistakes of the model.

## Results

Osteolytic lesions mostly affected the sub-regions beside and under the mandibular molars and the maxilla and other mandibular sub-regions were less commonly affected (Table 1). Well-defined lesions disproportionately affected the maxilla more and the coronoid and condyle less.

**Table 1:** Sub-regions affected by osteolytic lesions. Each osteolytic lesion could affect multiple sub-regions and a sub-region was counted at most once per patient

	Median	Paramedian	Angle	Corpus	Ramus	Coronoid	Condyle	Maxilla
Well-defined	21	36	55	90	25	0	2	31
Ill-defined	48	113	100	154	26	8	6	1
Total	69	149	155	244	51	8	8	32

Four different model architectures have been successfully trained to detect and classify osteolytic lesions from PRs. Among the validated models, those with transformer layers were more effective at detecting ill-defined lesions compared to well-defined lesions (Table 2). The most effective model architectures were Mask R-CNN with a Swin-Tiny backbone (macro-F1 = 0.844) and Mask DINO (macro-F1 = 0.830). In contrast, architectures without transformer layers were less effective, with Mask R-CNN using a ResNet-50 backbone achieving a macro-F1 score of 0.759, and YOLOv5 achieving a macro-F1 score of 0.681. These results demonstrate that incorporating transformer layers into the architecture consistently improves performance compared to models relying solely on CNNs.

**Table 2:** Classification metrics. The results from the five validation folds were aggregated before computing the metrics for PRs with well-defined and ill-defined osteolytic lesions. Best results are highlighted in bold. Sens = sensitivity, Spec = specificity

	**Well-defined**			**Ill-defined**			**Mean**		
**Model**	Sens	Spec	F1	Sens	Spec	F1	Sens	Spec	F1
Mask R-CNN + ResNet-50	0.642	0.935	0.697	0.840	0.925	0.822	0.741	0.930	0.759
Mask R-CNN + Swin-Tiny	**0.691**	**0.977**	**0.784**	**0.912**	0.962	**0.904**	**0.801**	**0.969**	**0.844**
Mask DINO + ResNet-50	0.685	0.969	0.769	0.884	**0.964**	0.891	0.784	0.966	0.830
YOLOv5-m	0.636	0.822	0.582	0.801	0.907	0.780	0.719	0.864	0.681

**Fig. 3:**
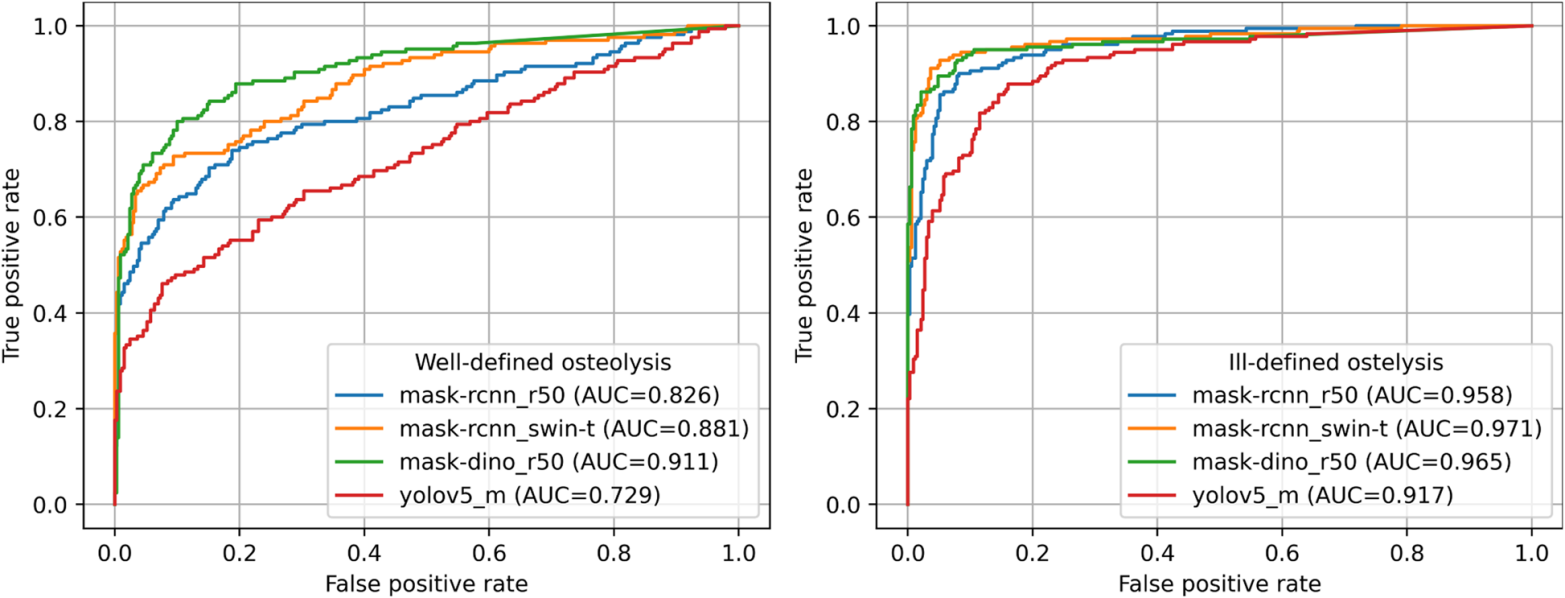
Receiver-operating-characteristics (ROC) curves for combinations of lesion pattern and model architecture. The curves are based on a comparison between PRs with a specific lesion pattern and control PRs. AUC = area under ROC curve

Additionally, the ROC curves, illustrating various combinations of lesion patterns and model architectures, demonstrated consistent outcomes. Among these, Mask R-CNN with Swin-Tiny backbone and Mask DINO were the most effective models, attaining AUC values of 0.881 and 0.911 for classifying well-defined PRs and AUC values of 0.971 and 0.965 for classifying ill-defined PRs, respectively (Fig. 3).

**Fig. 4:**
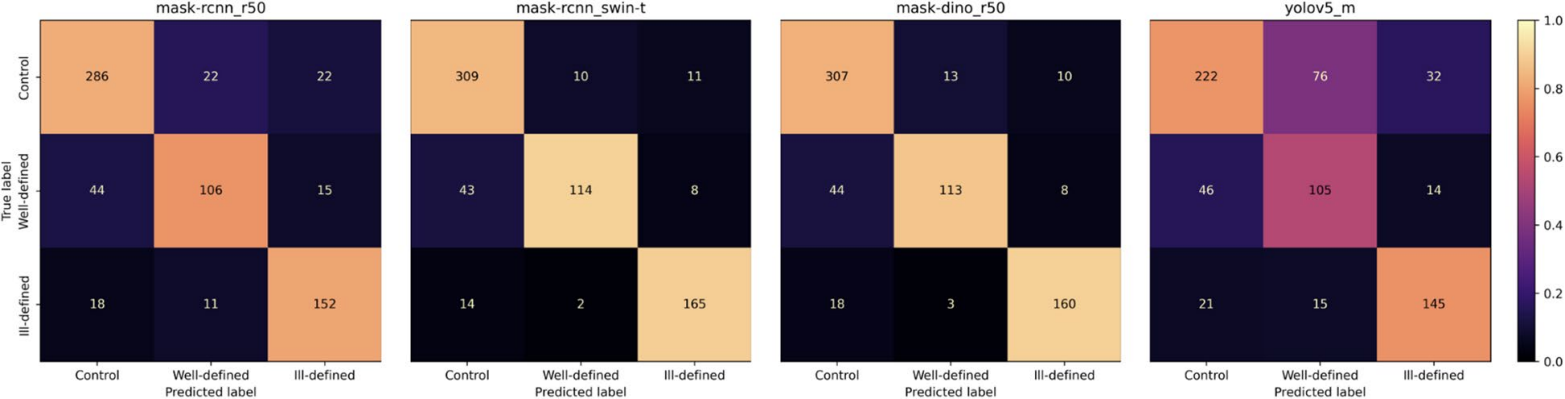
Confusion matrix for each model architecture illustrating image-level classification results. From left to right, the model architectures are Mask R-CNN with a ResNet-50 backbone, Mask R-CNN with a Swin-Tiny backbone, Mask DINO with a ResNet-50 backbone, and YOLOv5 with a medium backbone. The color bar is normalized according to the number of PRs per predicted label

**Fig. 5:**
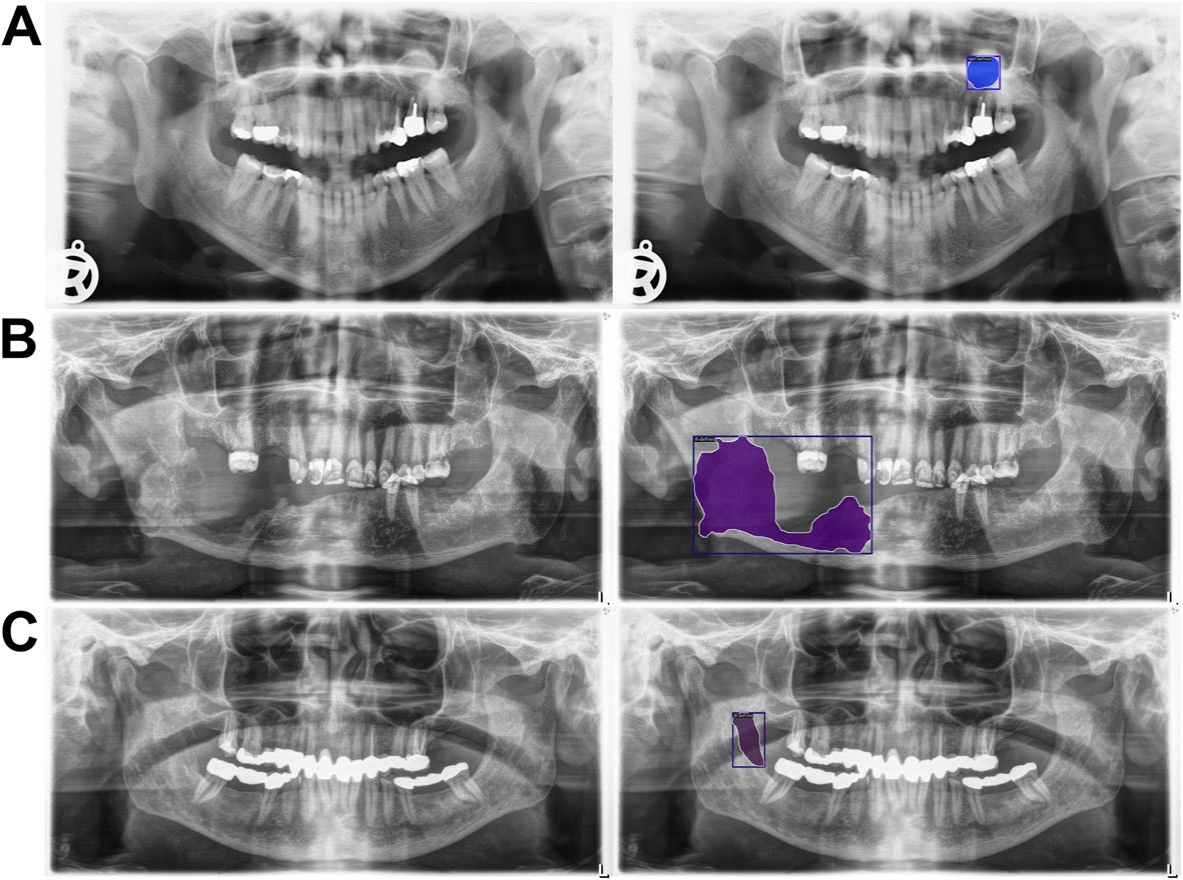
Examples of False-negative predictions. The panoramic radiographs (PRs) in the left column show cases where the most effective model—Mask R-CNN with a Swin-Tiny backbone—missed osteolytic lesions, which are displayed in the right column. In image **A**, the model missed a lesion in the upper jaw near the maxillary sinus. Image **B** shows a large osteolytic lesion in the mandibular angle that the model missed. In image **C**, a radiolucent band caused the model to miss the underlying lesion. These examples highlight common sources of false negatives, including radiographic artifacts and the maxillary sinus

**Fig. 6:**
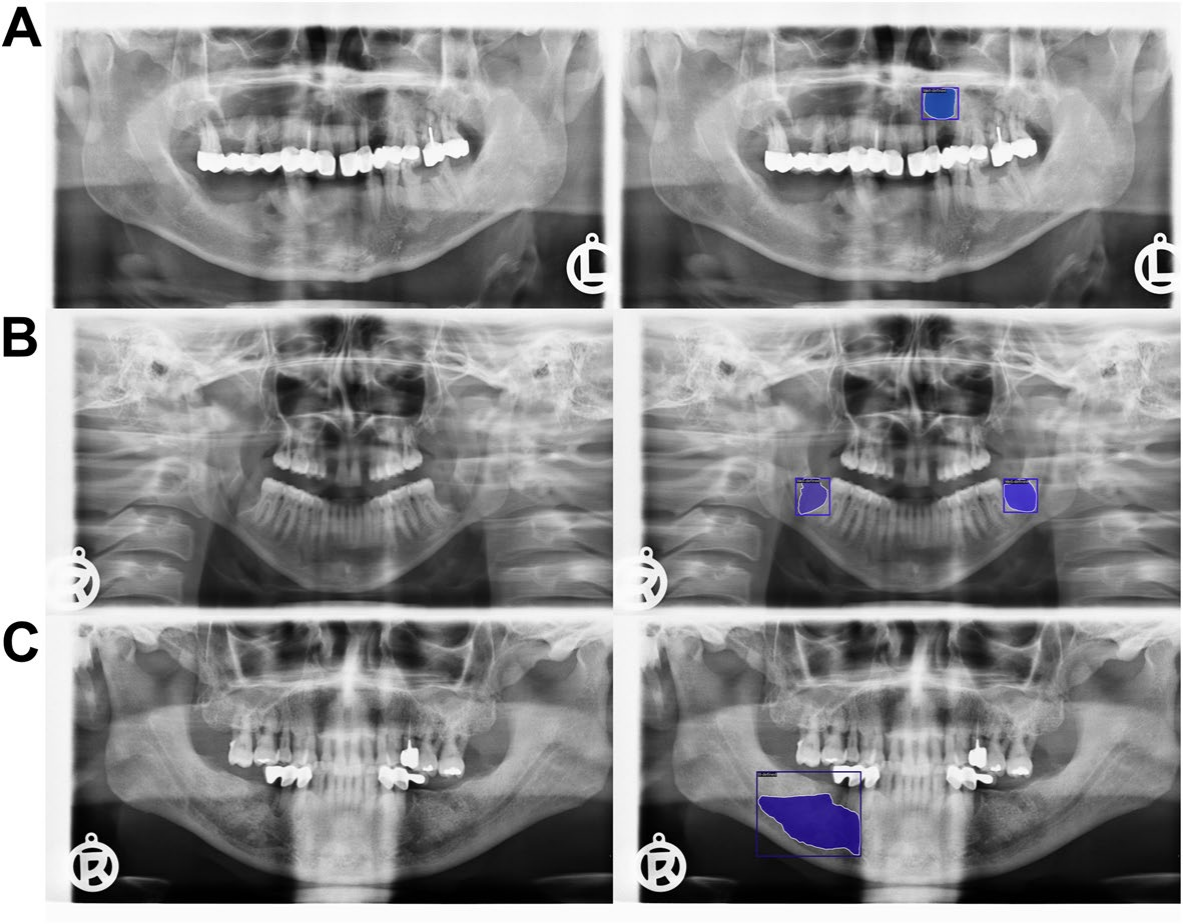
Examples of False-positive predictions. The panoramic radiographs (PRs) in the left column show cases where the most effective model—Mask R-CNN with a Swin-Tiny backbone—falsely predicted osteolytic lesions, as displayed in the right column. In image **A**, the model incorrectly identified a lesion in the upper jaw near the maxillary sinus. Image **B** shows false positive detections at sites of recent tooth extractions. In image **C**, an imaging artifact was mistakenly annotated as a lesion. These examples highlight common sources of false positives, including anatomical variations, extraction sites, and radiographic artifacts

**Fig. 7:**
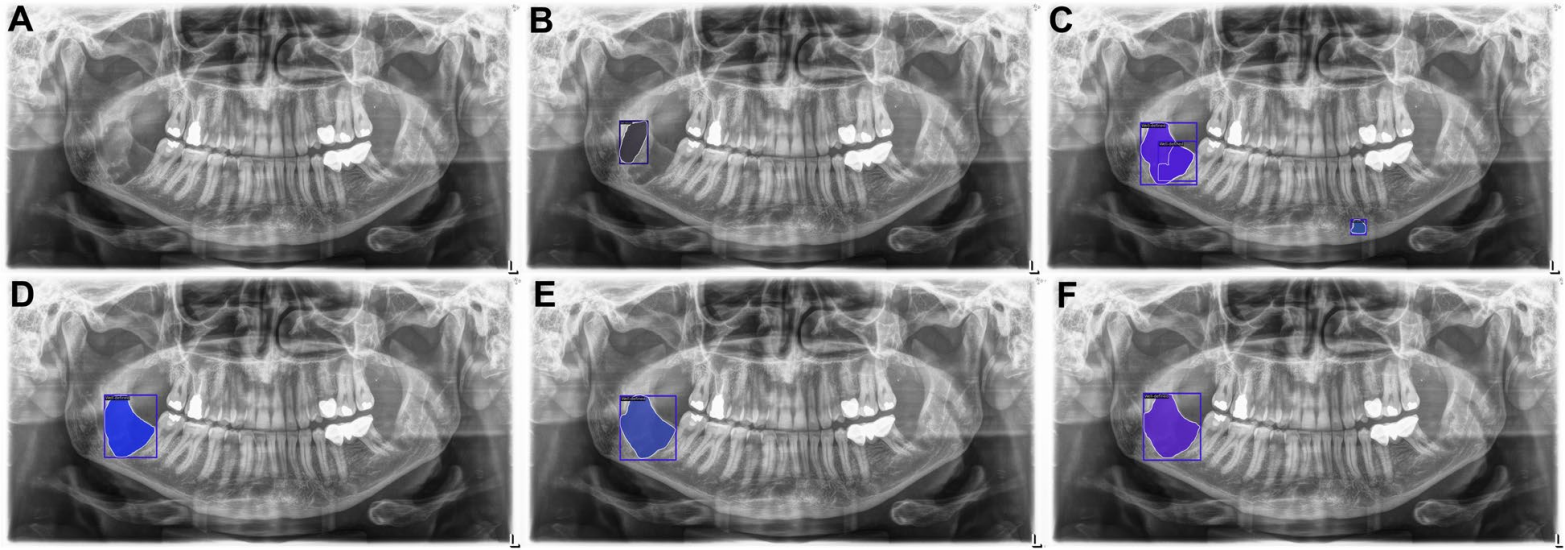
A panoramic radiograph (**A**) is shown with a well-defined lesion annotated (**B**). The predictions from four model architectures are shown: Mask R-CNN with a ResNet-50 backbone (**C**), Mask R-CNN with a Swin Transformer backbone (**D**), Mask DINO (**E**), and YOLOv5 (**F**)

**Fig. 8:**
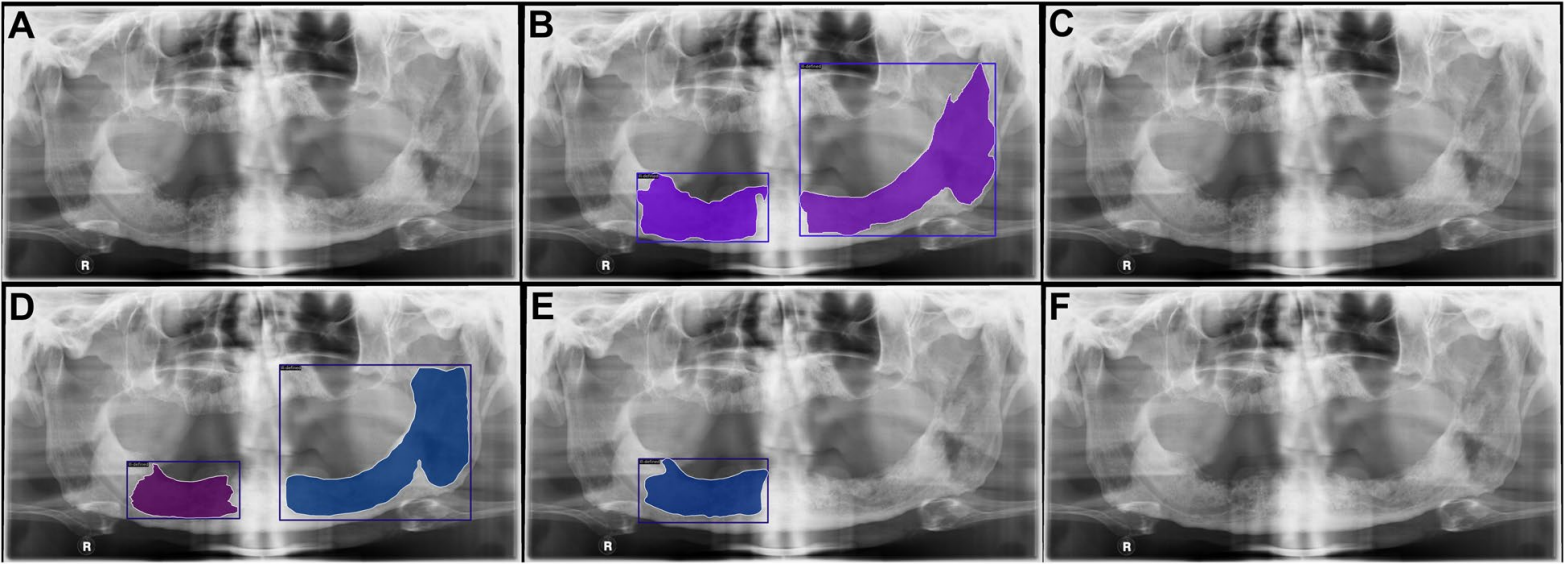
A panoramic radiograph (**A**) is shown with an ill-defined lesion annotated (**B**). The predictions from four model architectures are shown: Mask R-CNN with a ResNet-50 backbone (**C**), Mask R-CNN with a Swin Transformer backbone (**D**), Mask DINO (**E**), and YOLOv5 (**F**)

The confusion matrices show that more well-defined lesions were regarded as control compared to ill-defined lesions, as there were overall more well-defined lesions in our study sample (Fig. 4).Moreover, the qualitative analysis highlighted challenges in distinguishing between control PRs and PRs with well-defined lesions, especially in regions close to the maxillary sinus or following third molar extractions (Figs. 5 and 6). Additionally, it was observed that overprojections leading to large areas of relative radiolucency posed greater difficulty for the models in terms of detecting osteolytic lesions. Two PRs with results from all model architectures are shown in Figs. 7 and 8. The well-defined lesion in Fig. 7 is detected by all methods and Mask R-CNN with a ResNet-50 backbone falsely predicts a lesion. In comparison, the ill-defined lesion in Fig. 8 is only detected by Mask R-CNN with a Swin-Tiny backbone and Mask DINO, whereas the other methods missed the lesion.

## Discussion

The objective of this study was to detect osteolytic lesions in PRs and to categorize them as either well-defined or ill-defined using deep learning methods, specifically CNNs and vision transformers. Four distinct model architectures were implemented and trained for instance segmentation with the goal of predicting the presence of well-defined or ill-defined osteolytic lesions within a given PR. A comparison of the different model architectures revealed that transformer-based models outperformed their CNN-only counterparts in detecting osteolytic lesions**.** Mask R-CNN with a Swin-Tiny backbone achieved the highest overall performance, particularly for ill-defined lesions, highlighting the benefit of using transformer layers for capturing long-range dependencies in PRs. Mask DINO with a ResNet-50 backbone also performed strongly, benefiting from its end-to-end segmentation pipeline and attention mechanisms, despite having a CNN backbone. In contrast, Mask R-CNN with ResNet-50, while effective, showed lower sensitivity and F1-scores, particularly for well-defined lesions. YOLOv5**,** designed for real-time performance, demonstrated the lowest accuracy among all models, likely due to its one-stage architecture and limited ability to model complex spatial patterns in the image. These results underscore the importance of model architecture choice when designing AI systems for dental radiograph interpretation. This superiority was further confirmed by the ROC analysis, with Mask R-CNN (Swin-Tiny) and Mask DINO achieving AUC values of 0.881 and 0.911 for well-defined lesions and 0.971 and 0.965 for ill-defined lesions, respectively. These results underscore the advantage of transformer-based architectures in modelling long-range spatial dependencies within radiographs—an essential factor when analyzing complex anatomical patterns.

Prior studies have investigated the detection and classification of well-defined osteolytic lesions in PRs. Sivasundaram and Pandian [26] conducted a study involving 1171 real-time PRs, which were categorized into dentigerous, odontogenic and radicular cysts, as well as non-pathological PRs. They trained a classification model and employed morphological operations for segmenting detected cysts, achieving a sensitivity and specificity of 0.983 and 0.988, respectively. Another study annotated cyst-like radiolucent lesions with bounding boxes in 7,160 PRs and classified them based on their location [28]. They trained an object detection model for the detection of cyst-like lesions, achieving high specificity and sensitivity outcomes. Furthermore, several studies have focused on detecting and classifying malignant osteolytic lesions in whole-body CT scans. Faghani et al. segmented bony tissue and annotated lytic lesions in 2193 CT slices from 40 patients [22], achieving high sensitivity and specificity outcomes using a two-stage AI model.

In contrast, the present study is, to our knowledge, the first to apply vision transformer-based deep learning models for the pixel-level detection and classification of both well-defined and ill-defined osteolytic lesions in full PRs. The inclusion of ill-defined lesions, that are commonly associated with infections or malignancies, addresses a critical diagnostic need that has been largely underrepresented in existing AI research for dental imaging. Moreover, by evaluating and comparing multiple model architectures, our study not only identifies the most effective method but also provides a detailed analysis of how architectural components influence diagnostic accuracy.

From a clinical perspective, the implications of this work are significant. The proposed AI system can assist dentists by automatically highlighting suspicious regions in PRs during routine examinations. This form of decision support can be especially valuable for general practitioners who may lack extensive radiologic training, offering a second layer of scrutiny that promotes early detection of pathologies. Integration into existing clinical workflows—for example, as an overlay in radiographic software or PACS—would allow seamless, real-time interaction without requiring substantial changes in practice infrastructure.

The model’s performance in identifying ill-defined lesions, in particular, holds substantial clinical relevance, as these may indicate serious underlying conditions such as osteomyelitis or malignancy. With sensitivities exceeding 90% and specificities above 95% for the best-performing models, the rates of false positives and false negatives fall within acceptable clinical limits for use as a screening tool. Nonetheless, a careful examination of misclassifications revealed that false positives were most frequently found in anatomically complex areas such as the maxillary sinus or in regions with post-extraction bone remodelling. Conversely, false negatives typically involved small or poorly contrasted lesions, particularly in areas of dense bone or superimposed structures.

Although the model provides high-level performance, several limitations must be acknowledged. The system does not currently differentiate between various types of well-defined lesions, such as dentigerous cysts, radicular cysts, and ameloblastomas. Its role is best conceptualized as a tool to alert clinicians to areas of concern, prompting further investigation rather than offering definitive diagnoses. Additionally, despite a large dataset overall, the number of PRs containing osteolytic lesions (346 out of 6,404) remains limited, with a lesion prevalence higher than that seen in general dental populations. This selection bias may affect generalizability, and further validation using external, multicenter datasets is essential.

Future work should focus on enhancing the robustness and clinical applicability of the models by incorporating data from multiple institutions, integrating histopathological outcomes, and exploring hybrid architectures that combine radiographic imaging with patient metadata. Moreover, adding a pre-processing pipeline for tooth segmentation or anatomical landmark detection may improve model focus and reduce false predictions in anatomical regions.

## Conclusions

Promising deep learning models were developed using a vision transformer for the identification and categorization of osteolytic lesions in PRs. Among these models, Mask R-CNN with a Swin-Tiny backbone and Mask DINO were particularly effective. These findings underline the potential of vision transformer-based architectures in enhancing the AI-assisted detection and classification of osteolytic lesions, offering a significant improvement over traditional CNN-based models.

## Data Availability

The data used in this study can be made available from the corresponding author within the regulation boundaries for data protection.

## Abbreviations

PR: Panoramic radiograph
(CB)CT: (Cone-beam) computed tomography
CNN: Convolutional neural network
R-CNN: Region-based convolutional neural network
DINO: Self-distillation with no labels
AI: Artificial intelligence
IQR: Interquartile range
NMS: Non-maximum suppression
COCO: Common objects in context
ROC: Receiver operating characteristics
AUC: Area under the receiver-operating-characteristic curve
RPN: Region proposal network
ROI: Region of interest
IoU: Intersection over union
